# Design and Implementation of a SiC-Based VRFB Power Conditioning System

**DOI:** 10.3390/mi11121099

**Published:** 2020-12-12

**Authors:** Chao-Tsung Ma, Yi-Hung Tian

**Affiliations:** Department of Electrical Engineering, CEECS, National United University, Miaoli 36063, Taiwan; jakk226601735@yahoo.com.tw

**Keywords:** energy storage system, power conditioning system, silicon carbide, vanadium redox flow batteries

## Abstract

An energy storage system using secondary batteries combined with advanced power control schemes is considered the key technology for the sustainable development of renewable energy-based power generation and smart micro-grids. The performance of energy storage systems in practical application mainly depends on their power conditioning systems. This paper proposes a silicon carbide-based multifunctional power conditioning system for the vanadium redox flow battery. The proposed system is a two-stage circuit topology, including a three-phase grid-tie inverter that can perform four-quadrant control of active and reactive power and a bi-directional multi-channel direct current converter that is responsible for the fast charging and discharging control of the battery. To achieve the design objectives, i.e., high reliability, high efficiency, and high operational flexibility, silicon carbide-based switching devices, and advanced digital control schemes are used in the construction of a power conditioning system for the vanadium redox flow battery. This paper first describes the proposed system topologies and controller configurations and the design methods of controllers for each converter in detail, and then results from both simulation analyses and experimental tests on a 5 kVA hardware prototype are presented to verify the feasibility and effectiveness of the proposed system and the designed controllers.

## 1. Introduction

In recent years, the development of secure, low-carbon, and renewable energy sources and various smart micro-grid systems [1], power converter-based system compensating devices [2,3], advanced power converters using state-of-the-art wide-bandgap (WBG) switching devices, and digital-integrated intelligent control schemes [4,5] have become very popular research topics in the field of electric power and energy engineering. To best facilitate the above-mentioned technologies, various types of power converters are normally required [6,7,8,9,10,11], whose main components are semiconductor power switches and various system control units. To further enhance and optimize the performance of power converters, advanced semiconductor switches based on WBG materials, also known as third-generation semiconductor materials, such as gallium nitride (GaN) and silicon carbide (SiC), are emerging as very promising solutions [12,13]. It is well known that WBG materials offer superior characteristics over silicon in terms of band gap, electron mobility, electric breakdown field, saturated electron velocity, and thermal conductivity, which make WBG devices much desired for switching applications with high-voltage, -power, -temperature, and -frequency requirements. In particular, SiC devices with high-frequency switching capability and superior thermal conductivity are suitable for high-voltage and -power applications, while GaN has the highest bandgap, electron mobility, electric breakdown field, and saturated electron velocity, normally used in low- to mid-power systems [14]. In [15], the performance of a digitally controlled 2 kVA three-phase shunt-active power filter using GaN high electron mobility transistors was demonstrated for the first time. In addition, since decarbonization and green energy are two of the modern trends, renewable energy-based distributed power generation and on-line energy management systems have been intensively researched over the past decade. How to improve the performance and optimize the application of grid-level energy storage systems (ESSs) has also become one of the necessary technologies to promote the sustainable development of renewable power generation, active power distribution systems, and micro-grids. At present, practical electric energy storage technologies include pumped hydro system, compressed air energy storage, battery energy storage systems (BESS), flow battery, superconducting magnetic energy storage, flywheel, supercapacitor, etc. [16,17]. Among the above-mentioned grid-level ESSs, the vanadium redox flow battery (VRFB) has the advantages of independent and flexible design of output power and energy storage capacity, high energy conversion efficiency, safety, and low maintenance costs, which make it very suitable for a wide range of applications, such as distributed power generation optimization, energy management and integrated power quality control technology related applications [18,19]. In general, the performance of ESSs in practical application mainly depends on their power conditioning systems (PCS). The PCS topology required by the general grid-connected BESS can be divided into two categories: single-stage [20,21,22,23] and two-stage [24,25,26,27] according to the circuit architecture. The single-stage system is more suitable for high-voltage, high-capacity battery packs, while the two-stage circuit architecture usually includes a single-phase or three-phase direct current to alternative current (DC/AC) converter and a bi-directional direct current to direct current (DC/DC) power converter for matching with a wider range of battery pack voltage specifications, and enabling the realization of different charging and discharging strategies. In fact, various battery-based ESSs have been developed for a long time; however, most BESSs reported in the literature are based on some specific operation and control functions required by the system concerned and the system operating functions in this kind of BESS are quite limited and cannot be universal leading a very high system cost, long payback period, and serious lack of application flexibility. To improve the above-mentioned shortcomings and to achieve an advanced and versatile ESS, this paper proposes a SiC-based multifunctional PCS for the VRFB.

## 2. The VRFB System and the Proposed PCS Topology

**Figure 1:**
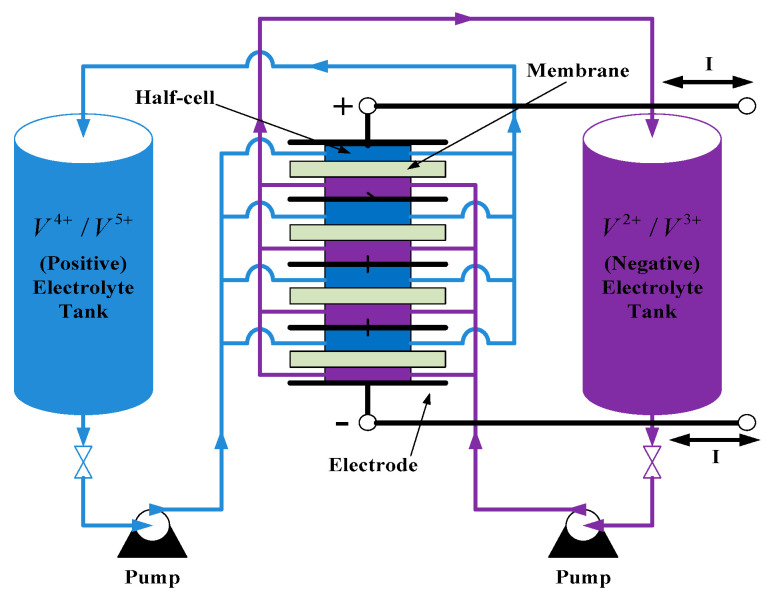
The system architecture of a vanadium redox flow battery (VRFB) [19].

**Figure 2:**
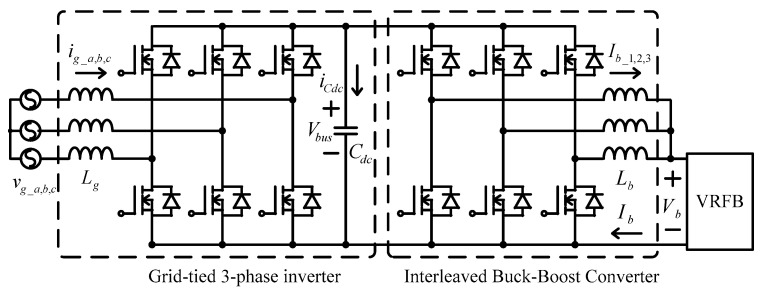
VRFB power conditioning system (PCS) circuit topology.

**Figure 3:**
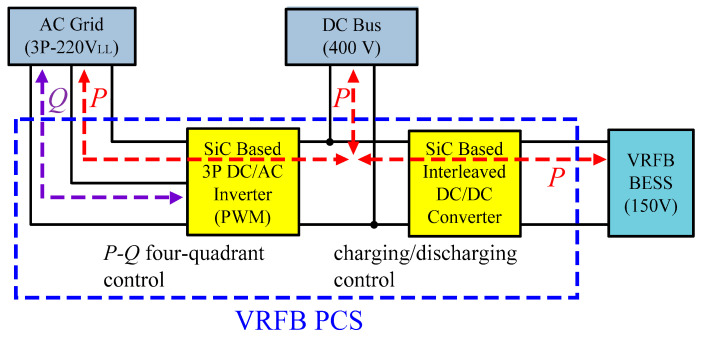
Possible active and reactive power flows in the proposed VRFB PCS.

The system architecture of a VRFB is shown in Figure 1. The two electrolytes, positive (V^4+^/V^5+^) and negative (V^2+^/V^3+^) electrolytes, in a VRFB are stored in different electrolyte storage tanks. During charging or discharging, the two electrolytes are separated by an isolation membrane, but selected ions are allowed to pass through the membrane forming a current path. The concentration and amount of electrolyte determine the system capacity of VRFB, the design specifications of electrodes determine the rated power of VRFB, and the number of single cells in series in the battery stack determines the maximum working voltage of VRFB. It is important to note that to achieve a cost-effective and high-efficiency design, the number of cells in series cannot be too high. This has resulted in a preferable lower system voltage. Considering this condition, a two-stage circuit topology is proposed for the VRFB PCS in this paper. In operation, both the DC/AC power converter and the interleaved multi-channel DC/DC converter are activated at the same time according to the due operating mode and system conditions. The detailed circuit architecture of the VRFB PCS proposed in this paper is shown in Figure 2, where the main function of the interleaved buck-boost converter, consisting of six SiC power semiconductor switches and inductors *L_b_*, is fast charging/discharging current command tracking. *L_b_* is used to filter out ripple components in the current caused by the switching of the semiconductor switch. As can be seen in Figure 2, the architecture consists of three parallel synchronous buck-boost converters, where the output switching signal of each converter is 120 degrees apart from another, offsetting each other’s ripples and reducing total output ripple. The left side of Figure 2 shows the grid-tied 3-phase inverter, whose main functions are DC bus voltage regulation via active power balancing control and system reactive power compensation via bi-directional reactive power tracking control. To provide a clear picture of the above-mentioned control functions, Figure 3 shows the possible active and reactive power flows in the proposed VRFB PCS.

## 3. Controller Design of VRFB PCS

The relevant system parameters and hardware specifications of the proposed VRFB PCS are shown in Table 1. Following in this section, the required mathematical model derivation and controller design will be carried out according to the specifications given in Table 1.

### 3.1. Grid-Tie Inverter Modeling and Design of Controllers

**Figure 4:**
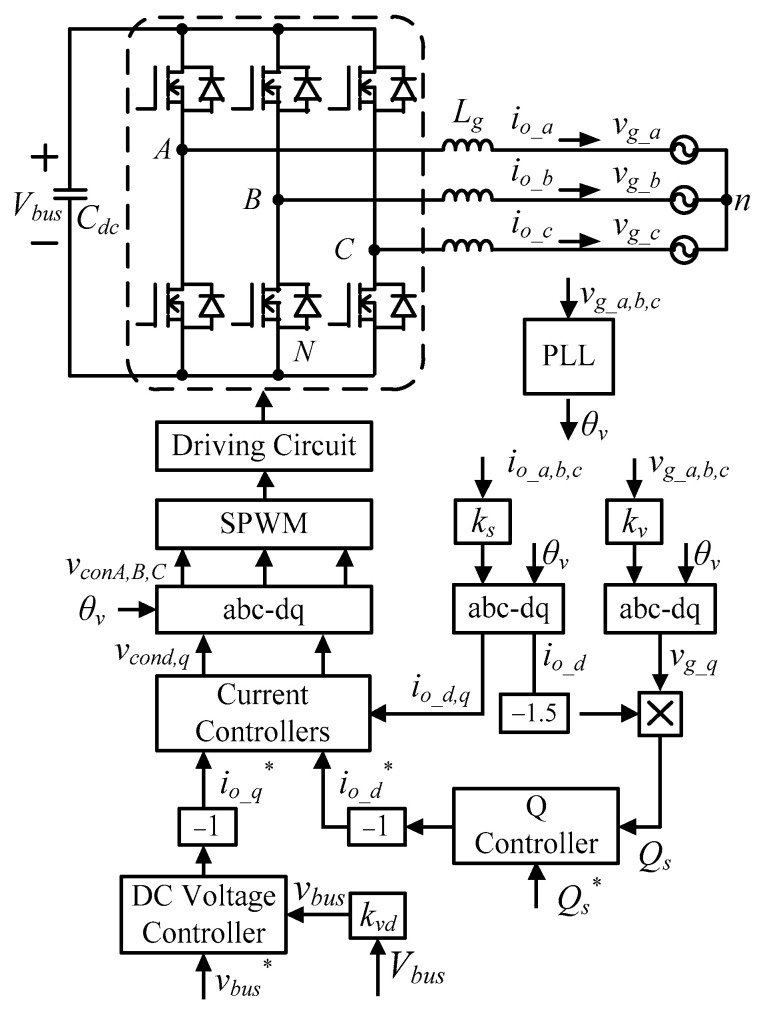
Overall control architecture of grid-tie inverter (* indicates commands).

To achieve a reliable control scheme, the grid-tie inverter adopts a dual-loop control architecture, where the inner loop controls inductor currents, and the outer loop controls DC bus voltage and AC-side reactive power. The overall control architecture is shown in Figure 4.

#### 3.1.1. Design of Inductor Current Controllers

The mathematical model of inverter’s inductor current in synchronous reference frame can be derived according to Figure 3:

(1) $$\begin{array}{l} \left[\begin{array}{l} L_{g}\frac{dI_{o\_d}}{dt}\\ L_{g}\frac{dI_{o\_q}}{dt}\\ L_{g}\frac{dI_{o\_0}}{dt} \end{array}\right]=K_{pwm}\left[\begin{array}{ccc} 1 & 0 & 0\\ 0 & 1 & 0\\ 0 & 0 & 1 \end{array}\right]\left[\begin{array}{l} v_{cond}\\ v_{conq}\\ v_{con0} \end{array}\right]-\left[\begin{array}{ccc} 1 & 0 & 0\\ 0 & 1 & 0\\ 0 & 0 & 1 \end{array}\right]\left[\begin{array}{l} V_{g\_d}\\ V_{g\_q}\\ V_{g\_0} \end{array}\right]-\\ \left[\begin{array}{ccc} 0 & \omega L_{g} & 0\\ -\omega L_{g} & 0 & 0\\ 0 & 0 & 0 \end{array}\right]\left[\begin{array}{l} I_{o\_d}\\ I_{o\_q}\\ I_{o\_0} \end{array}\right] \end{array}$$

**Figure 5:**
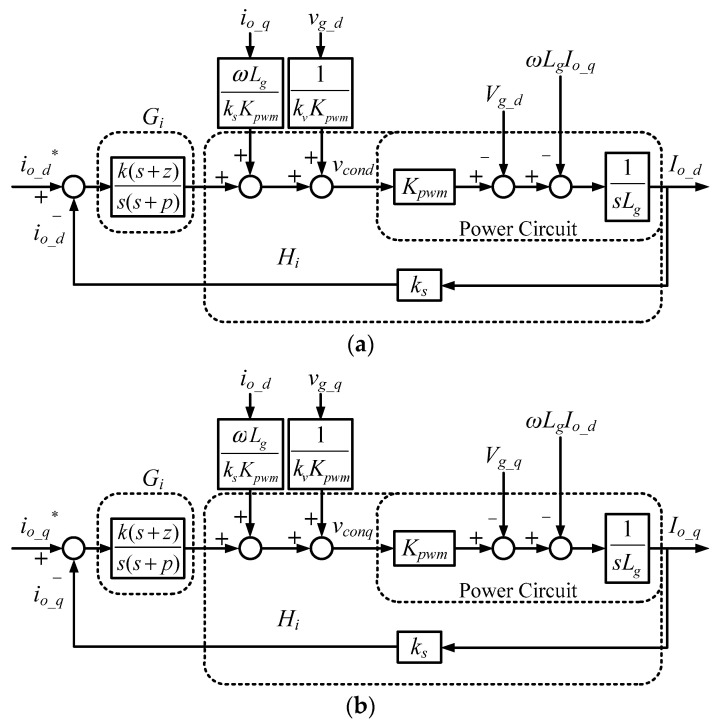
Inner inductor current control loops (* indicates commands): (**a**) d-axis; (**b**) q-axis.

In this paper, the Type II controller is used to control the inductor currents. Using (1) and the mathematical form of Type II controller, dq-axis inner inductor current control loops can be obtained, as shown in Figure 5.

The quantification design of the inner loop inductor current controller is as follows: choosing the crossover frequency *ω_i_* = 41,888 rad/s; zero = 8377.5 rad/s and pole = 136,282.2 rad/s, yielding the required Type II controller as follows:

(2) $$G_{i}(s)={\frac{3.953\times 10^{5}(s+8377.5)}{s(s+1.363\times 10^{5})}}^{}$$

**Figure 6:**
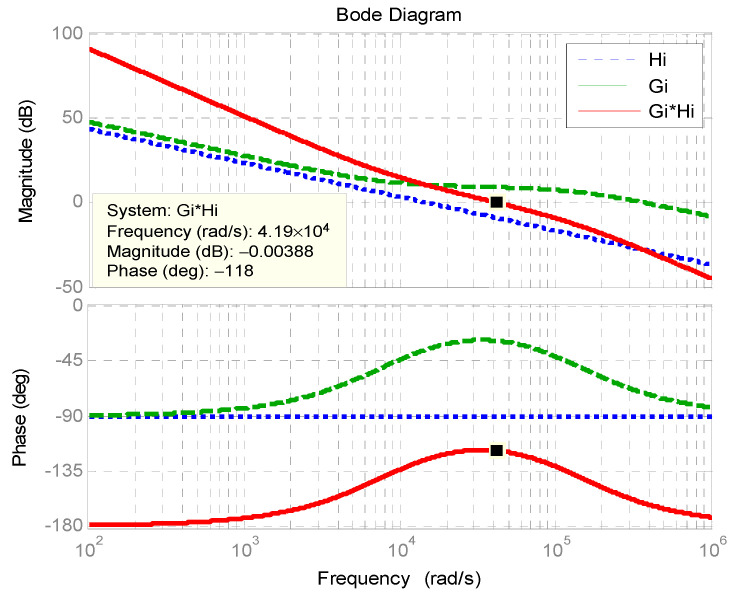
Bode plot of inner inductor current control loop.

Figure 6 shows the Bode plot of inner inductor current control loop. The phase margin is 62°.

#### 3.1.2. Design of the DC Bus Voltage Controller

**Figure 7:**
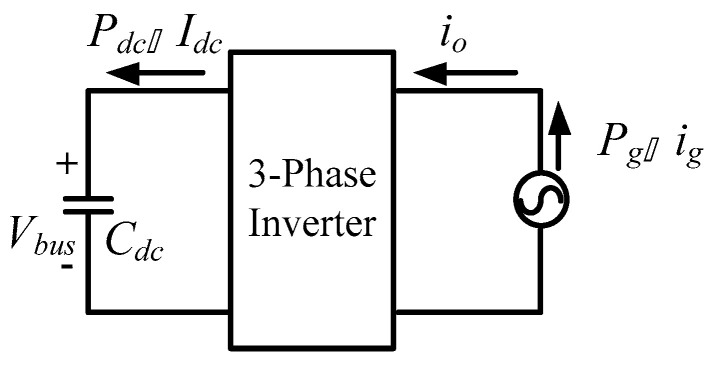
Equivalent circuit of direct current (DC) bus voltage loop.

Considering the steady-state operating point, the equivalent circuit of the DC bus voltage loop is as shown in Figure 7.

The mathematical model of DC bus voltage can be derived according to Figure 7:

(3) $$\frac{V_{bus}}{I_{o\_q}}=\frac{-k_{dc}}{sC_{dc}},k_{dc}=1.5\frac{V_{g\_q}}{V_{bus}}$$

**Figure 8:**
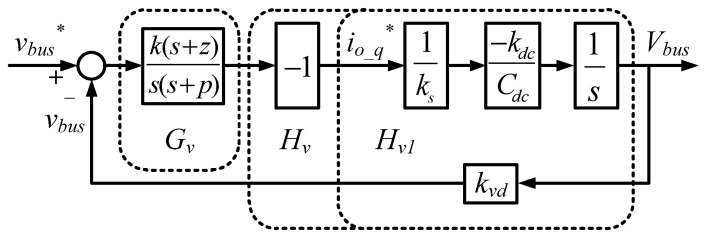
Outer DC bus voltage control loop (* indicates commands).

In this paper, the Type II controller is used to control the DC bus voltage, and thus outer DC bus voltage control loop can be obtained, as shown in Figure 8.

The quantification design of the DC bus voltage controller is as follows: choosing the crossover frequency *ω_v_* = 5235.9877 rad/s; zero = 523.598 rad/s; pole = 68,141.144 rad/s, yielding the required Type II controller as follows:

(4) $$G_{v}(s)=\frac{1.071\times 10^{7}(s+523.3426)}{s(s+6.814\times 10^{4})}$$

**Figure 9:**
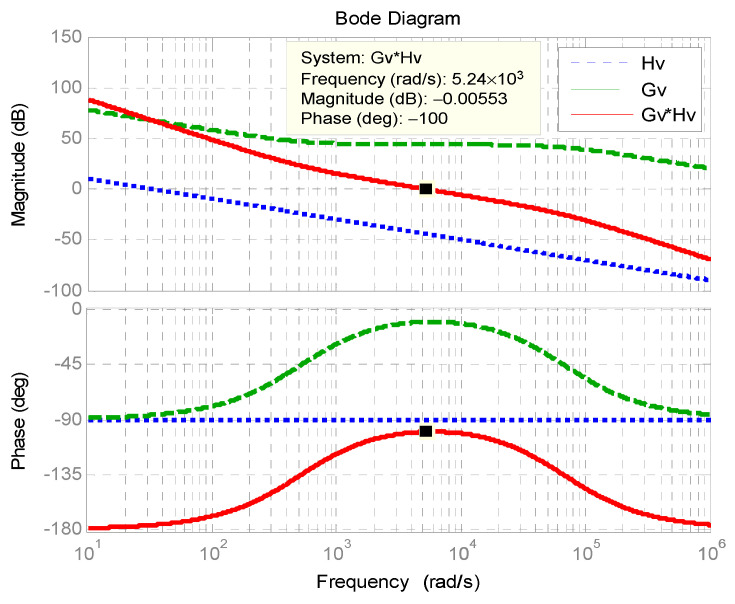
Bode plot of outer DC bus voltage control loop.

Figure 9 shows the Bode plot of outer DC bus voltage control loop. The phase margin is 80°.

#### 3.1.3. Design of Reactive Power Controller

The derivation of the AC-side reactive power controller in this paper takes the grid-side current flowing into the converter as positive:

(5) $$Q_{g}=-1.5\times V_{g\_q}\times I_{o\_d}$$

**Figure 10:**
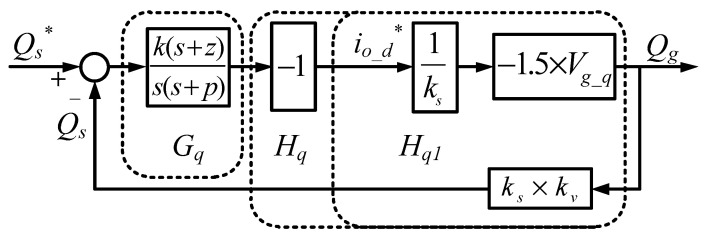
Outer reactive power control loop (* indicates commands).

In this case, the Type II controller is used to control the reactive power, and thus outer reactive power control loop can be obtained, as shown in Figure 10.

The quantification design of the reactive power controller is as follows: choosing the crossover frequency *ω_q_* = 3490.6585 rad/s; zero = 1745.329 rad/s; pole = 6981.317 rad/s, yielding the required Type II controller as follows:

(6) $$G_{v}(s)=\frac{8359(s+1745.4241)}{s(s+6981)}$$

**Figure 11:**
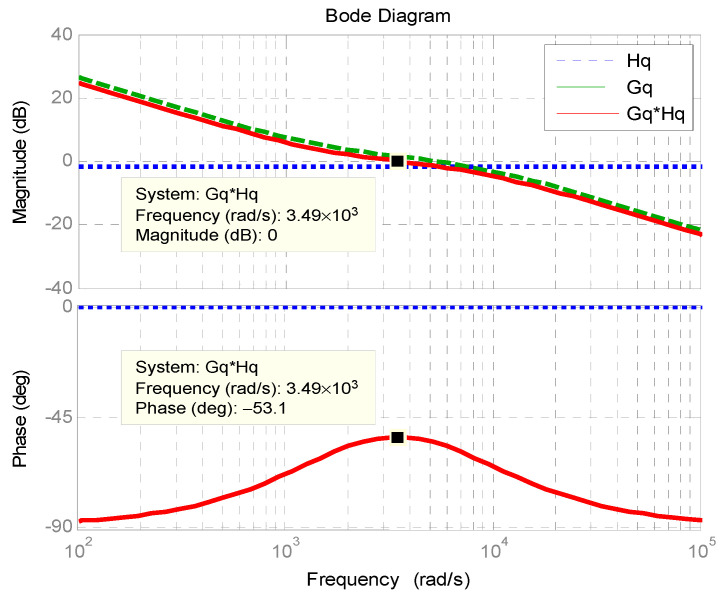
Bode plot of outer reactive power control loop.

Figure 11 shows the Bode plot of outer reactive power control loop. The phase margin is 127°.

### 3.2. Interleaved Buck-Boost Converter Controllers

**Figure 12:**
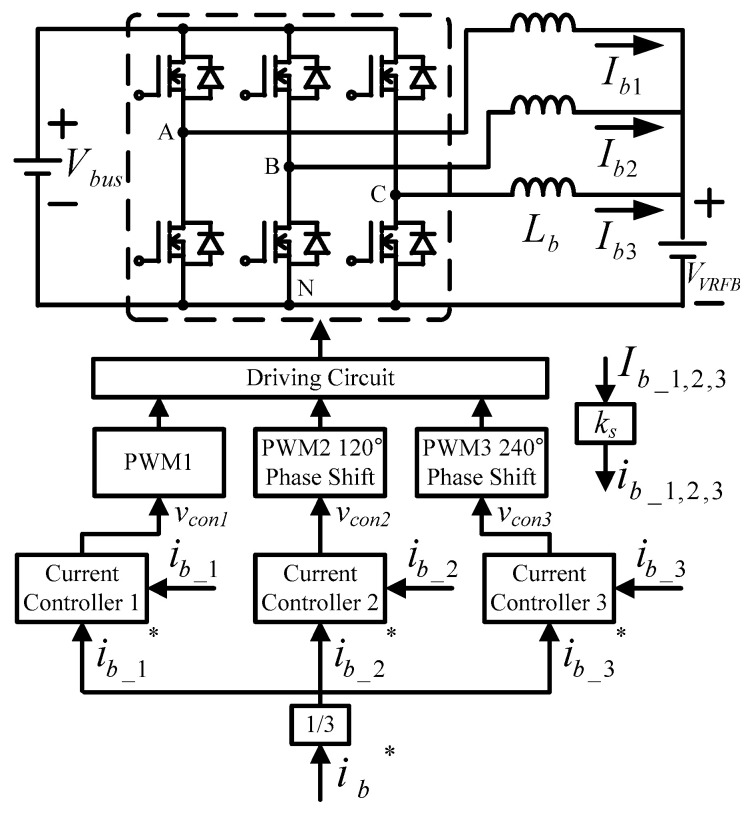
Overall control architecture of interleaved buck-boost converter (* indicates commands).

The interleaved buck-boost converter adopts a single-loop inductor current controller, and each channel is individually controlled and uses a different phase shift angle. The overall control architecture is shown in Figure 12.

#### Design of Inductor Current Controller

The proposed interleaved buck-boost converter is composed of multiple buck-boost converters, and its operating principle is the same as that of a single buck-boost converter. Therefore, only the controller design of a single buck-boost converter is illustrated. Taking leg A as an example, the mathematical model of the inductor current is as follows:

(7) $$L_{b1}\frac{di_{Lb1}}{dt}=v_{con1}K_{pwm}-V_{b},K_{pwm}=\frac{V_{bus}}{v_{tri}}$$

**Figure 13:**
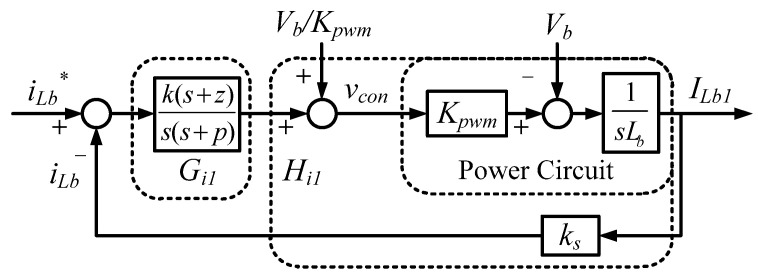
Buck-boost converter inductor current control loop (* indicates command).

In this control case, the Type II controller is again used to control the inductor current, and thus inductor current control loop can be obtained, as shown in Figure 13.

According to Figure 13, the transfer function of inductor current loop is as follows:

(8) $$H_{i1}(s)=K_{pwm}\times \frac{1}{sL_{b}}\times k_{s}$$

The quantification design of the inductor current controller is as follows: choosing the crossover frequency *ω_i_* = 39,270 rad/s; zero = 5167.1 rad/s; pole = 298,280 rad/s, yielding the required Type II controller as follows:

(9) $$G_{i}(s)=\frac{1.122\times 10^{6}(s+5167.1)}{s(s+2.9828\times 10^{5})}$$

**Figure 14:**
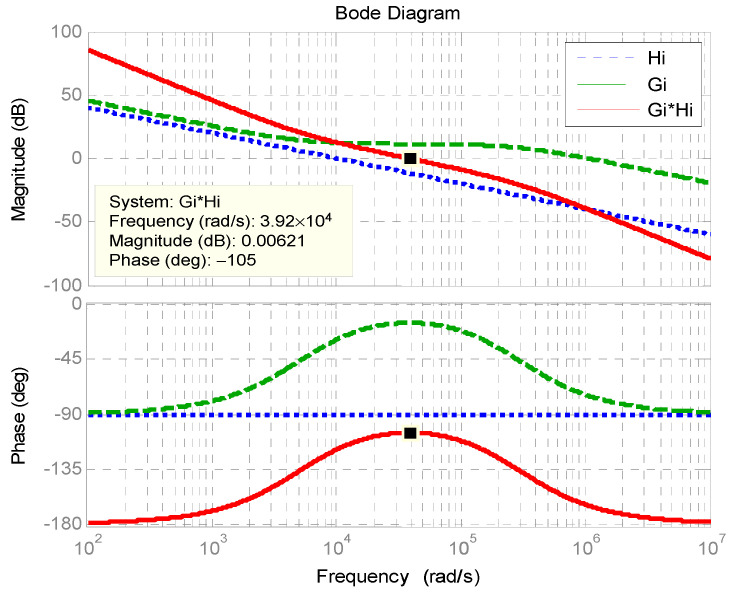
Bode plot of buck-boost converter inductor current control loop.

Figure 14 shows the Bode plot of the designed inductor current control loop. The phase margin is 75°.

## 4. Cases Simulation

### 4.1. Ramp-Up Procedure

**Figure 15:**
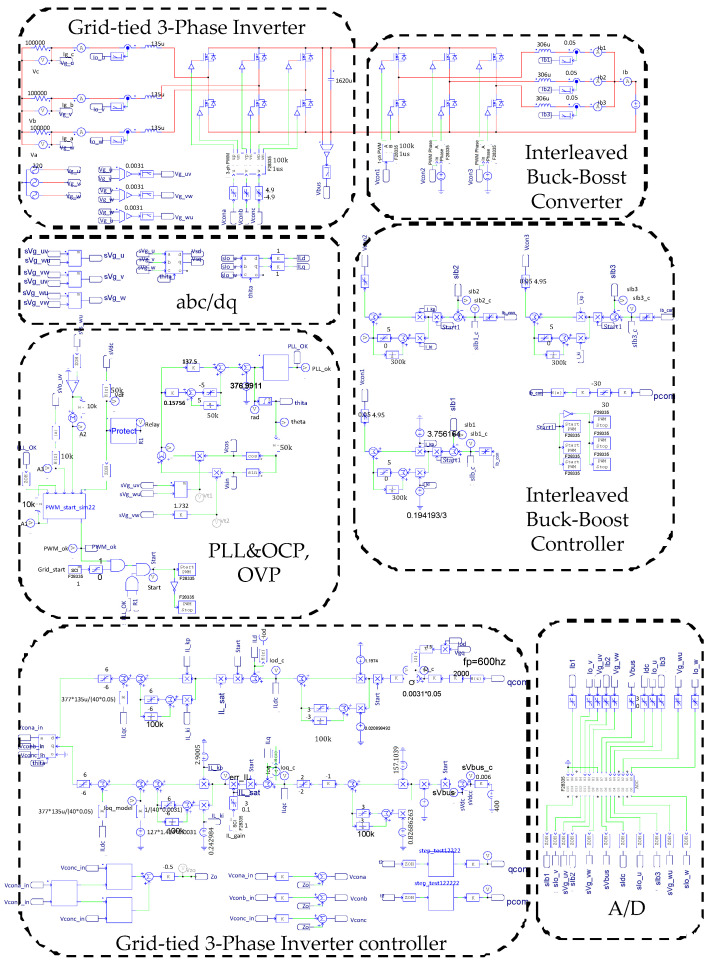
The power simulation software model of the proposed VRFB PCS.

**Figure 16:**
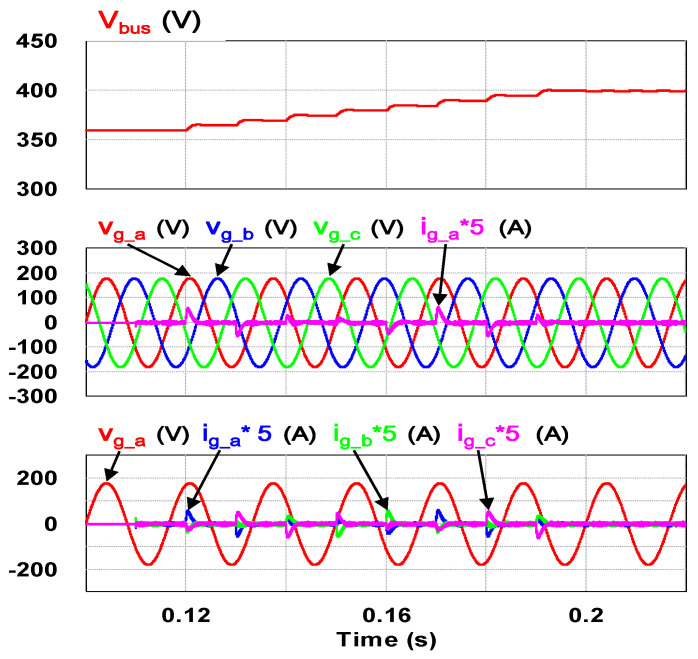
Waveforms of DC bus voltage (**top**), grid three-phase voltages and a-phase current (**mid**), grid a-phase voltage and three-phase currents (**bottom**).

To verify the correctness of the designed PCS controllers presented in the previous section, a software model of the proposed VRFB PCS is developed with power simulation software as shown in Figure 15. Two typical simulation cases, the ramp-up procedure and charging/discharging with four-quadrant P-Q control of the grid-tie inverter, are carried out in this study. Figure 16 shows the result of simulating ramp-up procedure of the system. This is to verify that the designed PCS can securely establish the required DC bus voltage of 400 V. As can be seen in Figure 16, after the grid-tie converter confirms the status of synchronization with the grid, the circuit starts to charge the DC bus capacitor slowly, and the rated DC bus voltage of PCS is boosted from 360 V and finally controlled at the target value of 400 V to complete the preparation of the system for various functional operations.

### 4.2. Charging/Discharging with P-Q Four-Quadrant Control of the Grid-Tie Inverter

**Figure 17:**
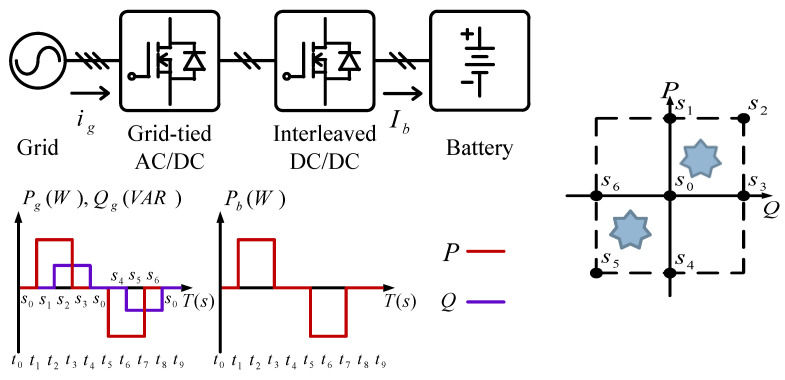
The schematic diagram of PCS operating in the 1st and 3rd quadrants.

**Figure 18:**
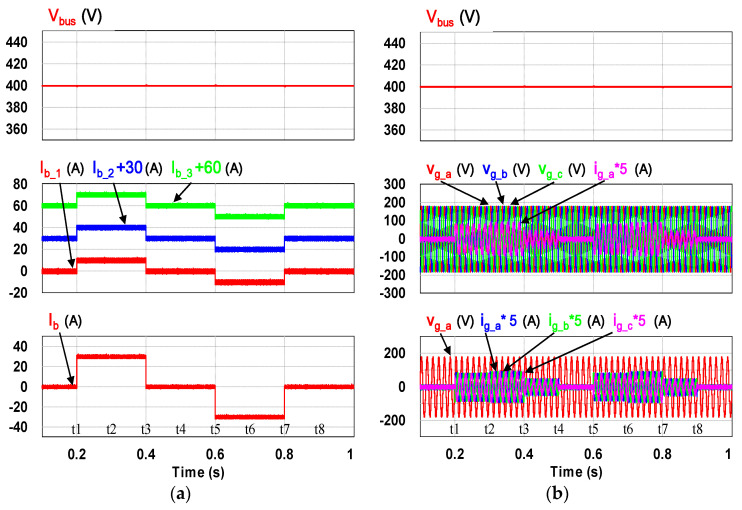
Waveforms of (**a**) interleaved buck-boost converter: DC bus voltage (**top**), interleaved inductor currents (**mid**), battery current (**bottom**) and (**b**) grid-tied inverter: DC bus voltage (**top**), grid three-phase voltages and a-phase current (**mid**), grid a-phase voltage and three-phase currents (**bottom**).

**Figure 19:**
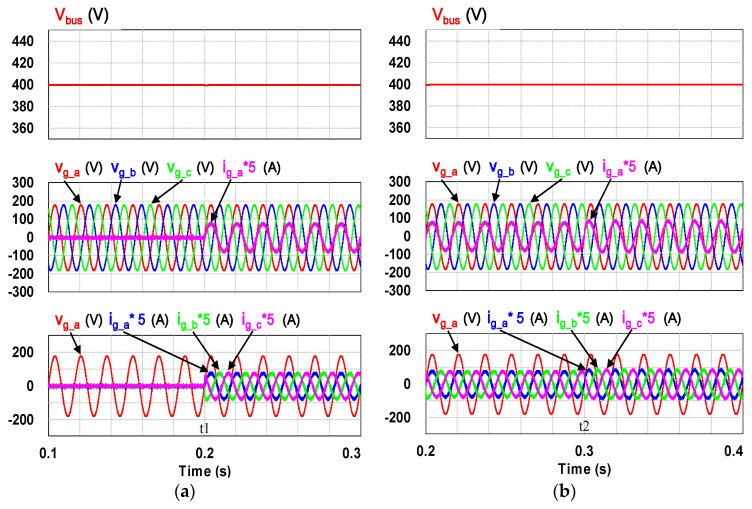
The detailed view of Figure 18b: (**a**) near t1; (**b**) near t2.

**Figure 20:**
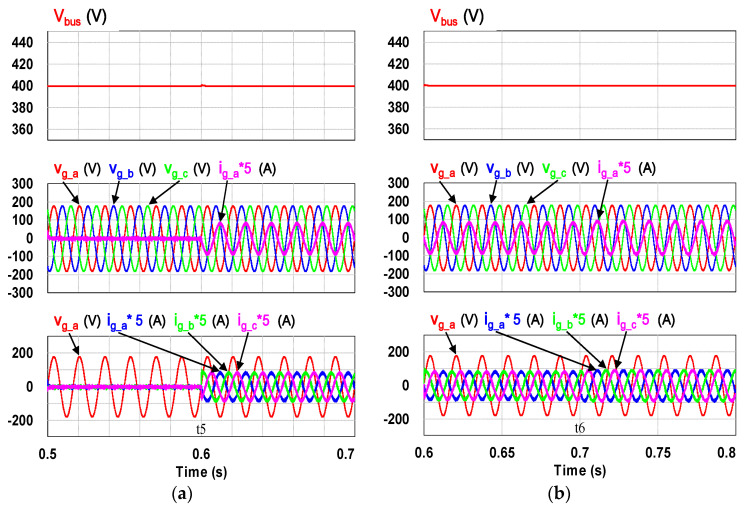
The detailed view of Figure 18b: (**a**) near t5; (**b**) near t6.

**Figure 21:**
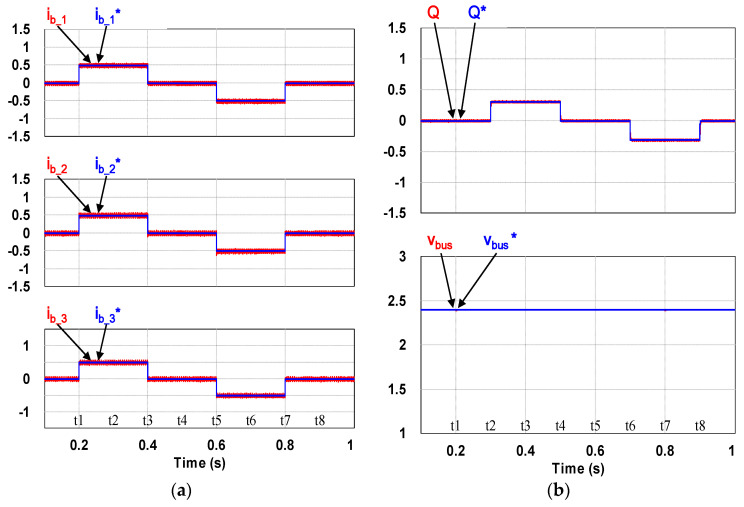
Waveforms of control commands and feedbacks: (**a**) three interleaved inductor currents; (**b**) reactive power (top), DC bus voltage (bottom).

**Figure 22:**
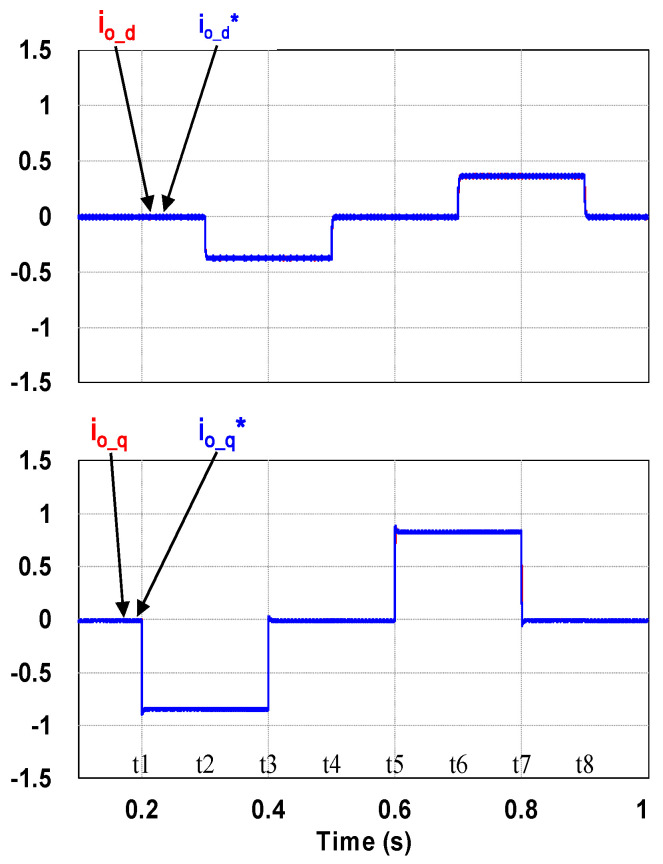
Waveforms of control commands and feedbacks: qd-axis inductor currents.

This case verifies simultaneous operation of the charging/discharging of VRFB and the function of reactive power regulation. In this operation mode, the charging/discharging current of the VRFB respectively corresponds to the positive and negative active power of the grid-tied inverter. With the independent control function of positive and negative reactive power regulation, a four-quadrant P-Q control is achieved by the grid-tied inverter. In this simulation case, the battery voltage = 150 V, a charging and discharging current command of ±30 A (equivalent to ±4.5 kW) and a ±2 kVAR reactive power command is arranged. Figure 17 shows the schematic diagram of PCS operating in the 1st and 3rd quadrants. Figure 18, Figure 19, Figure 20, Figure 21 and Figure 22 show a set of complete simulation results. As shown in Figure 18a, the three interleaved inductor currents are regulated evenly while the DC bus voltage is stably controlled at its rated value of 400 V. It can be clearly seen from Figure 19 and Figure 20, with the proposed direct current control scheme, the cross interference between active and reactive power of the grid-tied inverter is negligible. Figure 21 and Figure 22 show the tracking performance of the designed reactive power, charging and discharging controllers.

**Figure 23:**
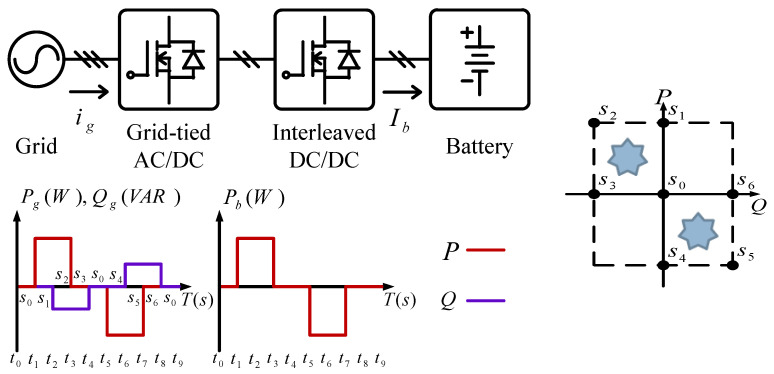
PCS operating in the 2nd and 4th quadrants.

**Figure 24:**
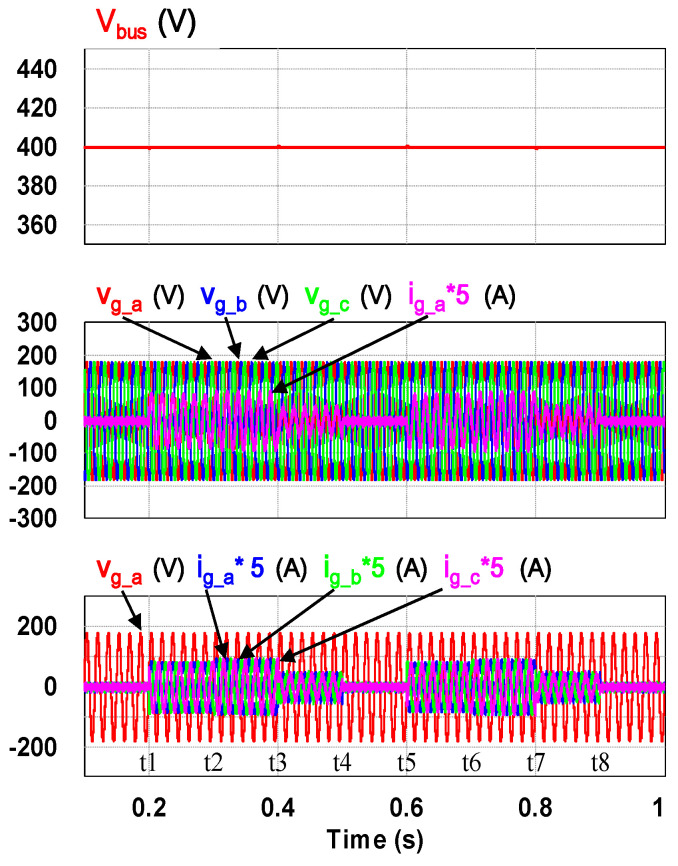
Waveforms of DC bus voltage (**top**), grid three-phase voltages and a-phase current (**mid**), grid a-phase voltage and three-phase currents (**bottom**).

**Figure 25:**
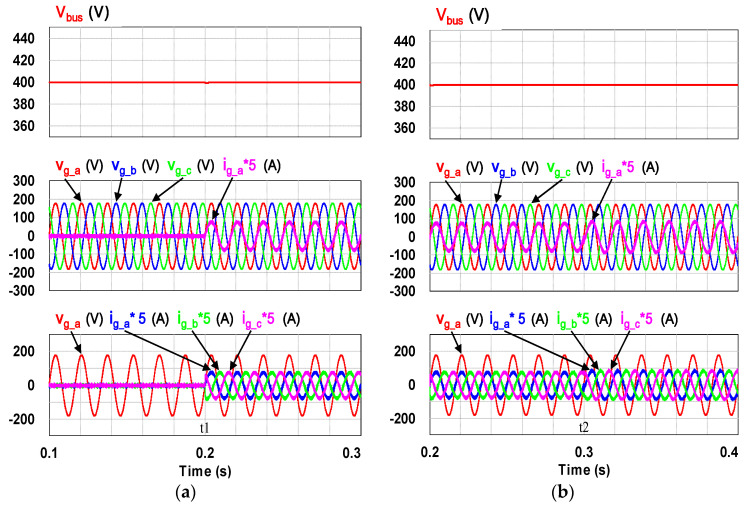
The detailed view of Figure 23: (**a**) near t1; (**b**) near t2.

**Figure 26:**
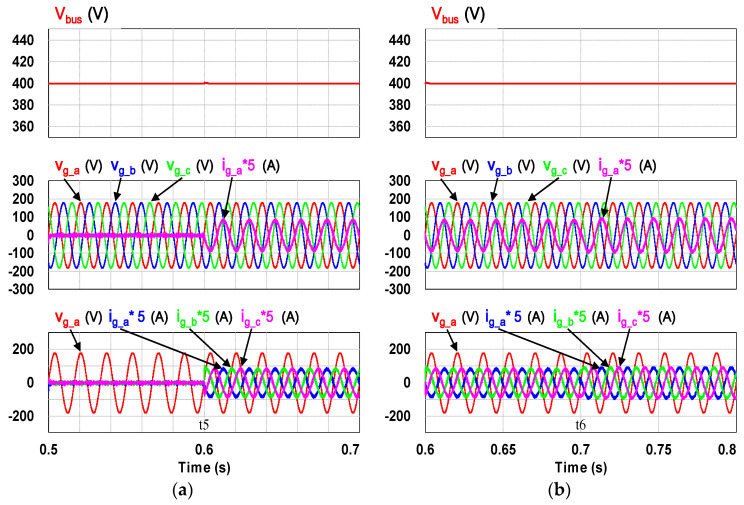
The detailed view of Figure 23: (**a**) near t5; (**b**) near t6.

**Figure 27:**
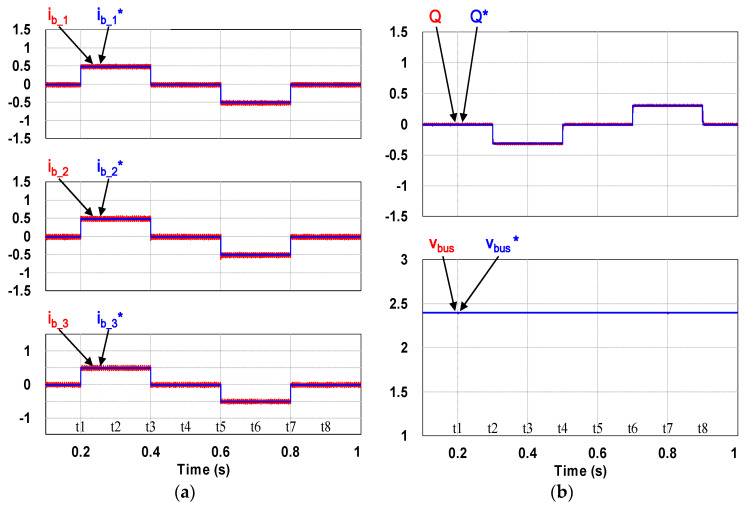
Waveforms of control commands and feedbacks: (**a**) three interleaved inductor currents; (**b**) reactive power (**top**), DC bus voltage (**bottom**).

**Figure 28:**
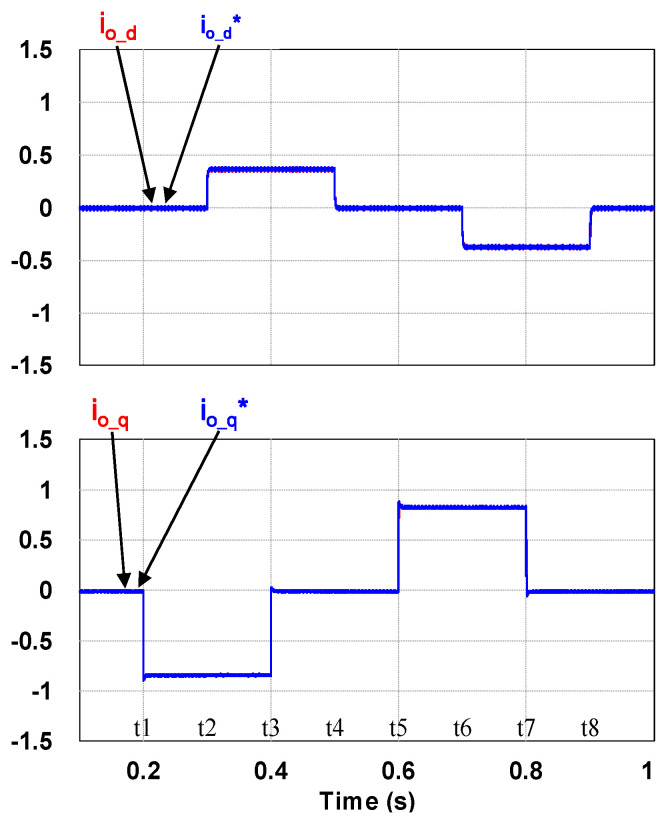
Waveforms of control commands and feedbacks: qd-axis inductor currents.

With the same operating condition as described previously, Figure 23 depicts a schematic diagram of PCS operating in the 2nd and 4th P-Q quadrants. Figure 24, Figure 25, Figure 26, Figure 27 and Figure 28 show a set of complete simulation results. In this case, with the same charging/discharging command, the current waveforms of interleaved buck-boost converter are identical to those shown in Figure 18a, so they are not shown in this case. As shown in Figure 24, during the charging and discharging operation of the battery the DC bus voltage is stably controlled at its rated value of 400 V with the designed voltage controller. It can be clearly seen from Figure 25 and Figure 26, with the proposed control scheme, the cross interference between active and reactive power of the grid-tied inverter is negligible. Figure 27 and Figure 28 verify the tracking performance of the designed reactive power, charging and discharging controllers working at different operating points.

## 5. Hardware Implementation and Test Results

**Figure 29:**
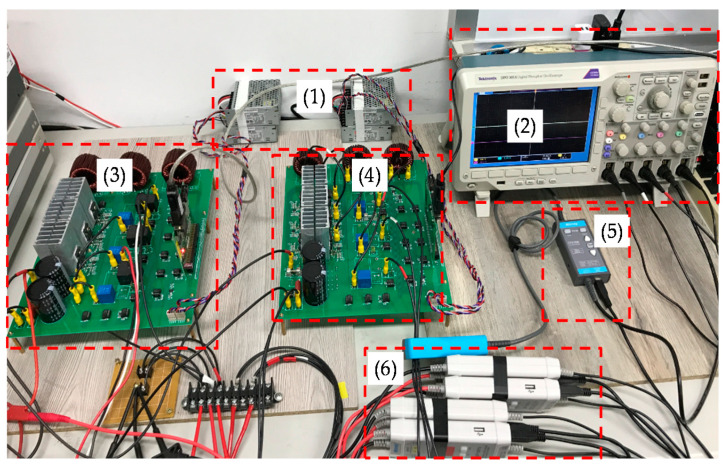
Photo of the constructed VRFB PCS hardware and the experimental platform.

**Figure 30:**
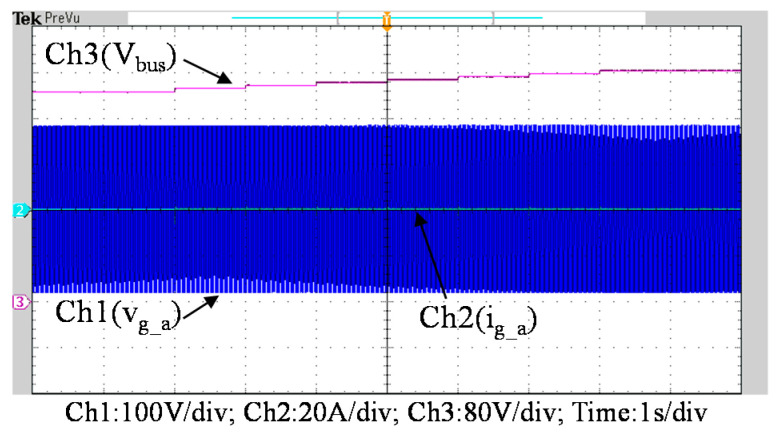
Waveforms of DC bus voltage (V_bus_), phase-a grid voltage (V_g_a_) and a-phase grid current (i_g_a_).

**Figure 31:**
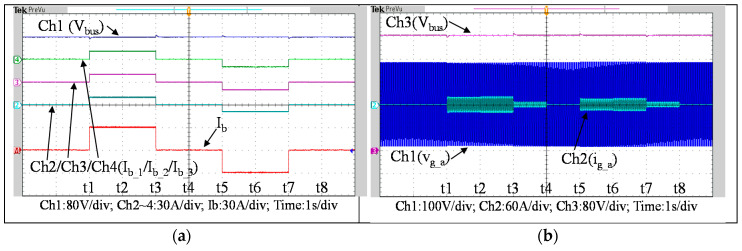
Waveforms of (**a**) interleaved buck-boost converter: DC bus voltage, interleaved inductor currents, and battery current, (**b**) DC bus voltage and the grid phase-a voltage and current.

**Figure 32:**
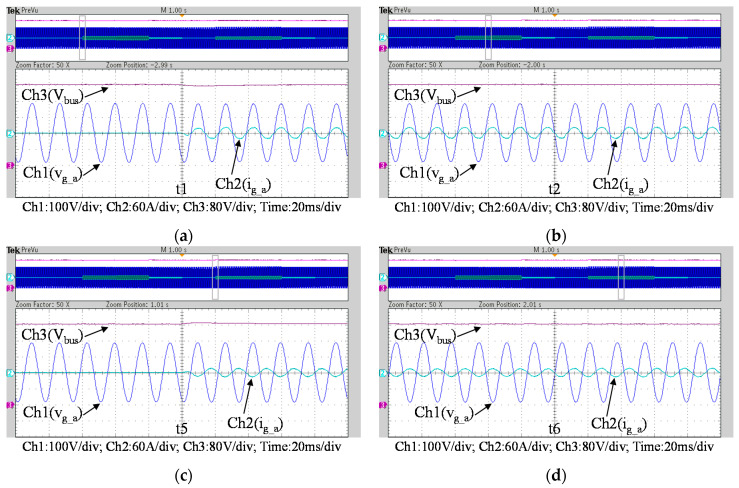
Detailed view of Figure 31b: (**a**) near t1; (**b**) near t2; (**c**) near t5; (**d**) near t6.

**Figure 33:**
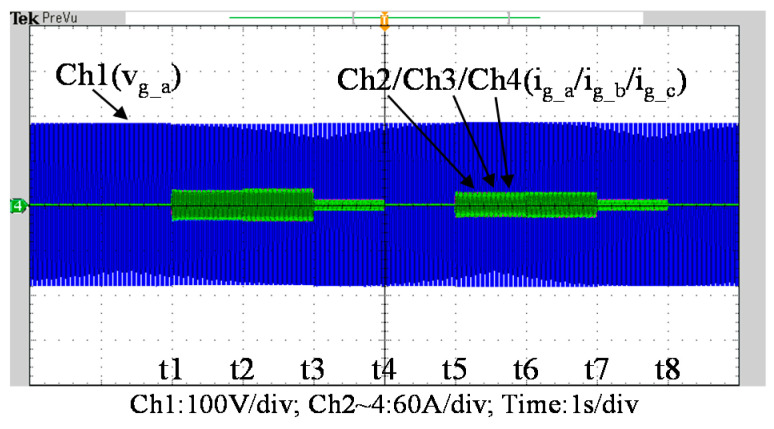
Waveforms of grid phase-a voltage and three-phase currents.

**Figure 34:**
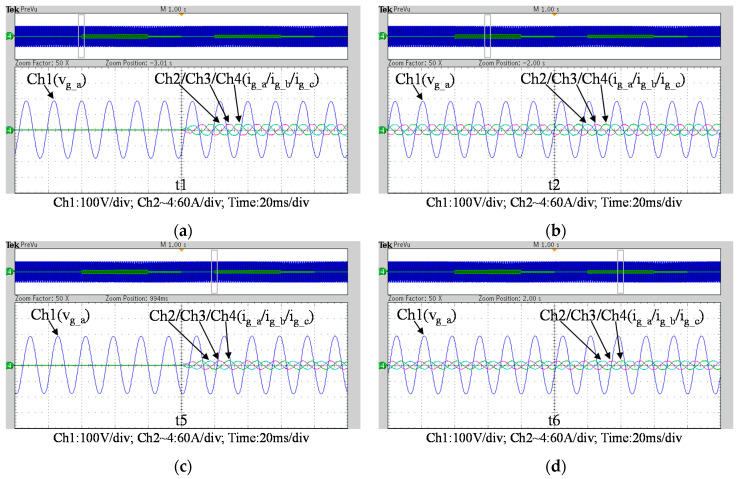
Detailed view of Figure 33: (**a**) near t1; (**b**) near t2; (**c**) near t5; (**d**) near t6.

To further verify the performance of the proposed VRFB PCS, a 5 kVA hardware experimental platform using SiC MOSFET is built according to the system specifications listed in Table 1 and the operating scenarios of the test cases are identical to that used in the simulated cases presented in the previous section. Figure 29 shows a photo of the constructed SiC-based VRFB PCS hardware system and the experimental platform, including (1) auxiliary power, (2) oscilloscope, (3) SiC-based grid-tie three-phase inverter, (4) SiC-based interleaved DC-DC buck-boost converter, (5) current probe, and (6) voltage probes. Figure 30 shows the test result of ramp-up procedure of the PCS hardware system. Figure 31, Figure 32, Figure 33 and Figure 34 show a set of experimental results of the proposed PCS operating in the 1st and 3rd quadrants. As can be seen in Figure 31, Figure 32, Figure 33 and Figure 34, the measured waveforms are very close to those obtained from simulation studies presented in the previous section. This has verified the feasibility and effectiveness of the proposed control schemes.

**Figure 35:**
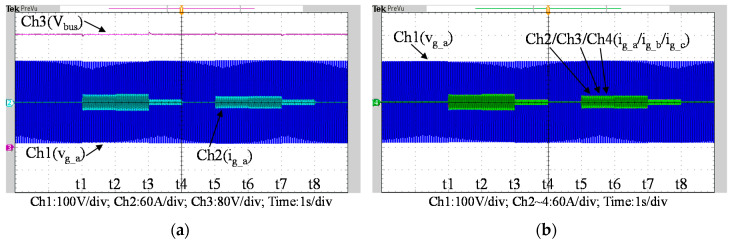
Waveforms of (**a**) DC bus and grid phase-a voltage and current; (**b**) three-phase currents.

**Figure 36:**
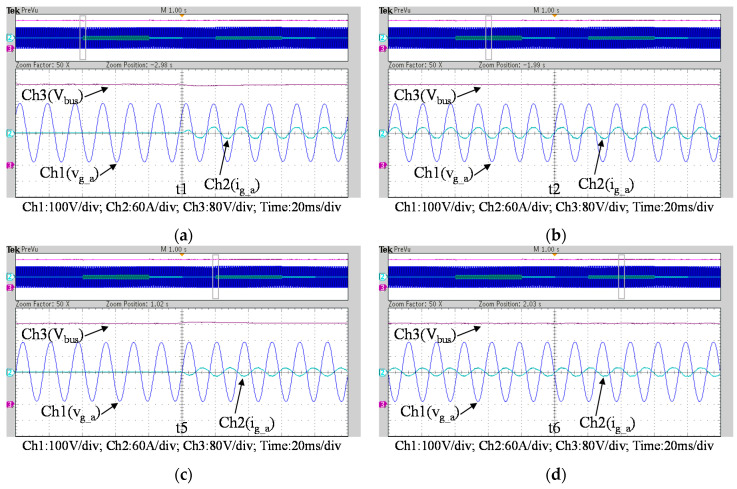
Detailed view of Figure 35a: (**a**) near t1; (**b**) near t2; (**c**) near t5; (**d**) near t6.

**Figure 37:**
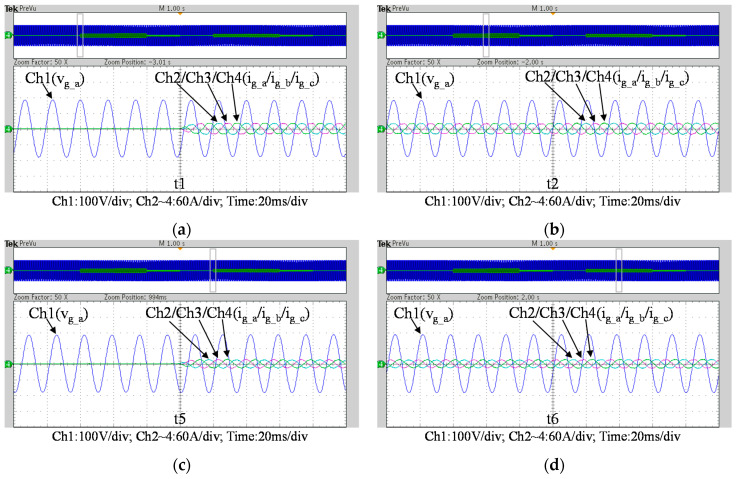
Detailed view of Figure 35b: (**a**) near t1; (**b**) near t2; (**c**) near t5; (**d**) near t6.

To fully verify the performance of the proposed SiC-based hardware system and control scheme, Figure 35 show a second set of experimental results, in which the proposed PCS is operating in the 2nd and 4th quadrants. As can be seen in Figure 35, Figure 36 and Figure 37, satisfactory performances of the proposed PCS grid-tied inverter and the interleaved buck-boost converter are achieved.

**Figure 38:**
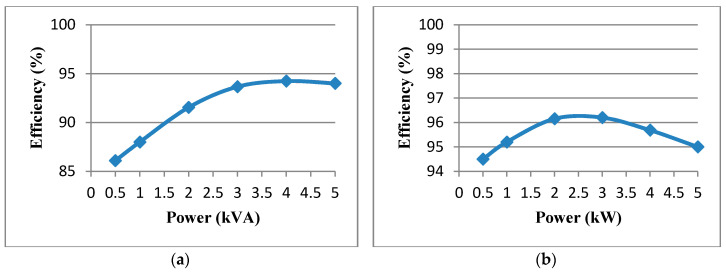
The efficiency analysis of the PCS: (**a**) grid-tied inverter, (**b**) interleaved buck-boost converter.

The efficiency test results of the proposed 5 kVA, SiC-based PCS’s grid-connected three-phase inverter and the 5 kW, 3-channel interleaved buck-boost converter are shown in Figure 38a,b respectively. The highest efficiency of the grid-tied three-phase inverter and 3-channel interleaved buck-boost converter system is measured as 94.1% at 80% system rated power and 96.3% at 60% system rated power, respectively.

## 6. Conclusions

It has been well accepted that the economic benefits of distributed power generation and micro-grids are multifaceted. For power users, the economic benefits lie in efficient use of energy, environmental protection, and reliable customized electrical energy services, while optimizing resource allocation and providing highly efficient energy management with operational flexibility are the main factors for achieving the economic benefits of micro-grids. However, with the addition of renewable power generations and various types of micro-grids in the power systems the complexity in system control and operation is significantly increased and certain compensating devices, e.g., ESSs integrated with advanced PCSs are urgently needed to be proposed and verified for feasibility. In this regard, this paper has proposed a SiC-based multifunctional PCS for the VRFB. In this study, it has been found that SiC switching devices with their excellent thermal and voltage capability can meet the requirement of a cost-effective design of grid-tied inverter system, in which the pulse width modulation technique can be used to reduce the hardware cost of PCS while improving system reliability. In this paper, the consideration of circuit topology and the detailed design steps of related controllers of the proposed SiC-based VRFB PCS have been fully addressed. The highest efficiency of the constructed SiC-based grid-tied three-phase inverter and 3-channel interleaved buck-boost converter system is measured to be 94.1% and 96.3% respectively. With the proposed PCS control schemes, four-quadrant control of active and reactive power and fast charging and discharging control of the VRFB have been achieved. Both simulation studies and experimental tests on a 5 kVA hardware prototype have verified the feasibility and overall performance of the proposed SiC-based VEFB PCS. It is worth noting that with the decoupled active and reactive power control capability and fast current command tracking feature the proposed VRFB PCS is expected to perform multiple system compensating functions, e.g., real-time support for renewable power generation, voltage and frequency support for micro-grids, and power quality improvement for power distribution systems.

## Figures and Tables

**Table 1:** Specifications of the proposed system.

Component	Item	Value
Grid	Three-phase line voltage	220 Vrms, V_LL_
	Voltage frequency	60 Hz
Interleaved buck-boost converter	Rated power	5 kW
	Number of channels	3
	VRFB pack voltage	136–153.6 V (48 cells)
	Switching frequency	100 kHz
	Switching device	SiC MOSFET
	Filter inductor	383 µH (20%)
	Current sensing factor	0.05 V/A
Grid-tie inverter	Rated power	5 kVA
	DC bus voltage	400 V
	Switching frequency	100 kHz
	Carrier voltage	5 V
	LPF	1st order (270 µH)
	DC bus capacitor	600 V/1620 µF
	DC voltage sensing factor	0.006 V/V
	AC voltage sensing factor	0.0031 V/V
	Current sensing factor	0.05 V/A
Controller	DSP	TI TMS320F28335
